# Housing Complexity Alters GFAP-Immunoreactive Astrocyte Morphology in the Rat Dentate Gyrus

**DOI:** 10.1155/2016/3928726

**Published:** 2016-02-18

**Authors:** Garrick Salois, Jeffrey S. Smith

**Affiliations:** The Brain Research Laboratory, Saginaw Valley State University, University Center, MI 48710, USA

## Abstract

Rats used in research are typically housed singly in cages with limited sensory stimulation. There is substantial evidence that housing rats in these conditions lead to numerous neuroanatomical and behavioral abnormalities. Alternatively, rats can be housed in an enriched environment in which rats are housed in groups and given room for exercise and exploration. Enriched environments result in considerable neuroplasticity in the rodent brain. In the dentate gyrus of the hippocampus, enriched environments evoke especially profound neural changes, including increases in the number of neurons and the number of dendritic spines. However, whether changes in astrocytes, a type of glia increasingly implicated in mediating neuroplasticity, are concurrent with these neural changes remains to be investigated. In order to assess morphological changes among astrocytes of the rat dentate gyrus, piSeeDB was used to optically clear 250 *μ*m sections of tissue labeled using GFAP immunohistochemistry. Confocal imaging and image analysis were then used to measure astrocyte morphology. Astrocytes from animals housed in EE demonstrated a reduced distance between filament branch points. Furthermore, the most complex astrocytes were significantly more complex among animals housed in EE compared to standard environments.

## 1. Introduction

Studies in neuroscience often employ animal models because of the similarity in structure and function between human nervous systems and the nervous systems of other animals. Furthermore, animal models allow changes in behavior to be compared directly to changes in neurophysiology, an ability that cannot be accomplished through any other means. For this reason, animal models are critical for our understanding of human nervous system function and disease. Despite the utility of animal models, results obtained from their use do not always translate to the human nervous system. This problem is exemplified by the near universal failure of clinical trials of treatments for numerous neurological diseases, including traumatic brain injury [1] and stroke [2], despite demonstrated efficacy in animal models. While many of the differences between humans and other animals are unavoidable, researchers must be careful to ensure their animal models are valid.

One of the most commonly used animal models in neuroscience is the rat. Rat nervous systems, like all other nervous systems, are capable of responding to changes in their environment by altering neurophysiology and, in turn, behavior [3]. This feat is accomplished through a process called neuroplasticity. Rodent nervous systems evolved to interact with a chaotic and complex natural ecological niche. Animal models used in the lab, like their wild counterparts, exhibit neurological adaptation to their environments [4]. However, rats used in research experience an environment dramatically different from the one to which their nervous systems have adapted. The standard housing environment (SE) used for rats in the laboratory involves a small plastic cage in which one or two rats can be housed [5]. This housing environment provides extremely limited sensory stimulation, little or no social interaction, and no opportunity for exercise. There is substantial evidence that these conditions profoundly affect the brain and behavior of animals, leading to abnormal neuroplasticity and pathological functioning [6, 7].

In contrast to the SE, enriched environments (EE) consist of a significantly larger enclosure with room for rats to be housed in groups [3]. As social animals, rats demonstrate increased species-normative behavior in the presence of other rats [8]. In addition to social stimulation, rats in enriched environments are exposed to novel stimuli in the form of regularly introduced species-appropriate toys and changes in the location of food sources [5]. Furthermore, the larger enclosure substantially increases the ability of the rat to explore and exercise [5]. In sum, enriched environments allow rats to experience a constantly changing environment that more closely approximates their natural environment.

Numerous studies have demonstrated significant differences in the behavior of animals housed in EE compared to SE housing. The different components of enriched environments, a larger enclosure, social interaction, and novel toys, have each been shown to be beneficial in isolation but provide synergistic benefits when combined [8]. Housing rodents in enriched environments have been demonstrated to reduce the abnormal stereotyping behaviors that frequently occur as a result of housing in SE [6]. Furthermore, studies have shown significantly improved performance among rats housed in EE on tests of learning and memory such as the Morris water maze and Barnes maze [9–11], tests of gross motor function such as the Rotor Rod [9, 10], and tests of anxiety and depression such as the Open Field Test, Elevated Plus Maze, and the Forced Swim Task [9, 10, 12].

Studies have also demonstrated significant changes in the neuroanatomy of animals housed in EE which may underlie the observed changes in behavior. Early studies of EE identified numerous changes in the brain following housing in EE, including changes in cortical depth and glia number [13, 14], brain weight [15, 16], and total DNA content [16]. A brain region which undergoes significant structural remodeling in response to EE is the hippocampus [17]. The hippocampus is critically involved in learning and memory [18] and in maintaining a neural representation of extrapersonal space [19]. Studies of hippocampal plasticity in response to EE have generally focused on mechanisms of neural adaptation. For example, studies have demonstrated that the hippocampus responds to EE with changes in patterns of neurotransmitter receptor expression [20], as well as increasing dendritic complexity [21], spine density [22], and neurogenesis [11, 23].

Evidence suggests that glia are a fundamental factor in modulating neural plasticity. The most numerous cell in the brain [24], the astrocyte, is a glial cell critical in mediating central nervous system homeostasis through several mechanisms. Astrocytes are known to make contacts with both synapses and vasculature [25–27]. Astrocytic end-feet engulf the gaps between endothelial cells of the vasculature and alter their permeability, thus forming an integral component of the blood brain barrier [28, 29]. The ability of astrocytes to junction neural activity with vascular glucose influx is the basis for BOLD (blood oxygenation level dependent) signal used by fMRI [26]. Astrocytes are also critically involved in the brain's response to insult, such as stroke or traumatic brain injury. Following injury, astrocytes migrate to the site of injury and become hypertrophic, forming a glial scar which limits the spread of inflammation and apoptosis to surrounding intact tissue but also impedes the ability of axons to reinnervate damaged tissue [30–34]. Furthermore, astrocytes are capable of dynamically altering the properties of neurons in both the short and long term. In the short term, astrocytes are capable of altering concentrations of ions and neurotransmitter at the synapse [26], as well as metabolizing glucose to lactate, the primary energy source used by neurons [35]. In the long term, astrocytes may influence neural plasticity through control of spine dynamics [36–39] and neurotransmitter receptor expression at the synapse [20, 40, 41]. Recent studies have also demonstrated that release of the NMDA receptor coagonist D-serine by astrocytes is required for long-term potentiation in the hippocampus and prefrontal cortex [42–46]. In the dentate gyrus of the hippocampus, astrocytes actively modulate the differentiation, growth, survival, and integration of newborn neurons [47, 48]. Astrocytes have also shown to be structurally dynamic, with their processes demonstrating surprising motility in response to synaptic activity within their domains [36, 49]. In the visual cortex, GFAP-immunoreactive astrocytes demonstrated significant increases in number and GFAP expression in response to EE [50]. Thus, physiological changes in astrocytes in response to EE are likely to be coincident with changes in neurons.

Differences in neurophysiology that occur due to housing environment may confound animal studies of nervous system function. In the case of astrocytes, significant alterations in physiology have been observed in all disease models in which they have been studied, including Alzheimer's Disease, Parkinson's Disease, and epilepsy [51–61]. For this reason, it is critical to understand the physiological changes that occur in the brain in response to the housing environment of the animal model used. The present study was designed to determine whether hippocampal astrocytes undergo gross morphological changes in response to rearing in enriched environments. Measures of morphological complexity, such as number of filament branches, total filament length, number of terminal points, and number of Sholl intersections, were used to assess the possibility that astrocytes may be altered in an experience-dependent manner. It was hypothesized that, due to the crucial role of astrocytes in neural function, EE would result in an overall increase in the number and complexity of astrocytes in the dentate gyrus.

## 2. Methods

All brain samples used in this study were pseudorandomly selected from sham animals used in a study conducted by Jacqmain, Nudi, Fluharty, and Smith, 2014, on the effects of environmental enrichment on recovery from medial frontal cortex contusion. Eight samples were used, with four samples from animals housed in standard environments and four samples from animals housed in enriched environments. All procedures were approved prior to experimentation by the Saginaw Valley State University Institutional Animal Care and Use Committee.

### 2.1. Previous Study Procedures

#### 2.1.1. Animals

Samples were selected from a cohort of 113 male Long-Evans rats (Charles River, Portage, MI) received on PND 25 weighing from 51 g to 75 g. On arrival animals were randomly assigned to either an individual standard laboratory cage (SE) or an enriched environment (EE) with a total of 10 rats per cage. Animals housed in the EE condition were transferred to EE cages with a total of 6 rats at PND 115.

#### 2.1.2. Housing Conditions

In each housing condition, animals were provided* ad libitum* access to food and water and were kept on a 12-hour reverse day/night cycle. Handling and behavior testing were conducted during the dark cycle. All rats were handled daily for 5 minutes per rat to acclimate them to human contact. Cell-sorb (Fangman Specialties, OH) was used as bedding for all cages and all rats were fed with rodent chow (Zeigler Bros, Inc., PN).

The SE condition consisted of a standard plastic laboratory cage (Alternative Design, Siloam Springs, AR) measuring 26.0 cm wide, 47.0 cm long, and 20.3 cm high. Rats were permanently given water and rodent chow through the top of the cage. One rat was housed per SE cage.

The EE condition consisted of a large plastic bin (Freedom Breeders #44, California) measuring 152.5 cm wide, 61 cm long, and 21.5 cm tall. Rodent chow was provided by a well placed along the width of the cage. Species-appropriate toys were added to EE cages, including PVC pipes, cardboard tubes, plastic shelters, wooden blocks, and chew toys. The toys were rearranged daily during handling and were changed during cleaning every three days. 10 animals were housed per cage until PND 115, at which point rats were housed in groups of six per EE enclosure.

#### 2.1.3. Euthanization and Tissue Handling

On postsurgery day 28 (PND 143) rats were deeply anesthetized with 5% isoflurane. Following absent eye-blink and tail pinch reflexes, rats were intraperitoneally injected with Euthasol (pentobarbital, 392 mg/kg). Rats were then transcardially perfused with 500 mL 0.9% phosphate buffered saline, followed by 500 mL 10% buffered formalin. The rats were decapitated and the brains were extracted. The left hemisphere from each brain was placed in 25 mL of 10% formalin overnight. The brains were then dehydrated and embedded in paraffin wax on plastic cartridges using a Tissue-Tek III vacuum infiltration tissue processor (IMEB Inc., San Marco, CA) and a paraffin-embedding console (Miles Scientific, Fergus Falls, MN). Samples were then stored at RT until they were used in the present study.

### 2.2. piSeeDB

Fluorescent immunohistochemistry and optical clearing techniques were used to allow imaging of cellular morphology in the dentate gyrus. SeeDB [62], a recent optical clearing technique, uses Fructose in PBS to match the refractive index of scatter of cellular membranes, enhancing tissue transparency. However, SeeDB has been reported to be ineffective with immunohistochemistry because it does not facilitate antibody penetrance [62]. Furthermore, SeeDB was not designed for use with paraffinized samples. The SeeDB procedure was modified in order to facilitate effective immunohistological labeling in paraffinized samples, resulting in a new procedure called piSeeDB. Our results indicate that a relatively simple modification to the SeeDB protocol whereby samples were exposed to a freeze/thaw cycle increased antibody penetrance and allowed imaging to depths of up to 2 mm [10].

#### 2.2.1. Deparaffinization

Paraffin-embedded left hemispheres were placed in a 40°C water bath for fifteen minutes prior to sectioning. The first 250 *μ*m section from each sample was collected and placed in a vacuum oven (Sheldon Manufacturing, Cornelius, OR). Samples were heated to 60°C with 15 inches' Hg vacuum for 8 hours. Tissue samples were then placed in a Tissue-Tek III tissue processor which was used to alternate pressure and vacuum with 50°C xylene for 1 hour. Sections were then dehydrated using ascending concentrations of ethanol.

#### 2.2.2. Freeze/Thaw

Recent work has established a method of freeze/thaw cycling that significantly improves immunostaining compared to other methods such as antigen retrieval when used with thick tissue samples [63]. Samples were placed in a sealed conical tube following dehydration. Samples were then placed in a freezer at −80°C for 30 minutes. Samples were then brought to room temperature for 30 minutes. This procedure was repeated four times for a total of four hours. Tissue was then rehydrated using descending concentrations of ethanol. Tissue was then rotated in a conical tube with 50 mL of 10% PBS and 1% Triton X-100 (PBS-T) at 36°C overnight.

#### 2.2.3. Immunohistochemistry

Tissue was placed in a twelve-well plate with 1.25 mL of DaVinci Green (Biocare Medical, Concord, CA) with 1% Triton X-100 (12.5 *μ*L) and incubated at 36°C for 24 hours. Tissue was then rinsed with PBS-T for 15 minutes. In order to block binding of endogenous IgG, samples were then incubated at 36°C in 1.25 mL of Rodent Block Rat (Biocare Medical, Concord, CA) for 24 hours. Tissue was then rotated in a 50 mL conical tube of PBS-T for three 30-minute washes with fresh PBS-T used for each wash.

Antibodies were added to 1.25 mL of DaVinci Green. Newborn neurons were labeled using 3.12 *μ*L of guinea pig polyclonal anti-doublecortin (1 : 100; Bioss Inc., Woburn, MA), astrocyte filaments were labeled using 12.5 *μ*L mouse monoclonal anti-GFAP conjugated with Cy3 (1 : 100; Abcam, Cambridge, MA), and neural cell bodies were labeled using 12.5 *μ*L rabbit monoclonal anti-NeuN conjugated with AlexaFluor 647 (1 : 100; Abcam, Cambridge, MA). Tissue samples were then incubated at 36°C in the primary antibody solution for 24 hours. Tissue was flipped after 12 hours had elapsed. Tissue was then rotated again in a 50 mL conical tube of PBS-T for three 30-minute washes. Tissue was then incubated at 36°C in DAPI solution (Biocare Medical, Concord, CA) for 6 hours. The tissue was then rinsed a final time with three PBS-T washes.

#### 2.2.4. SeeDB

**Figure 1:**
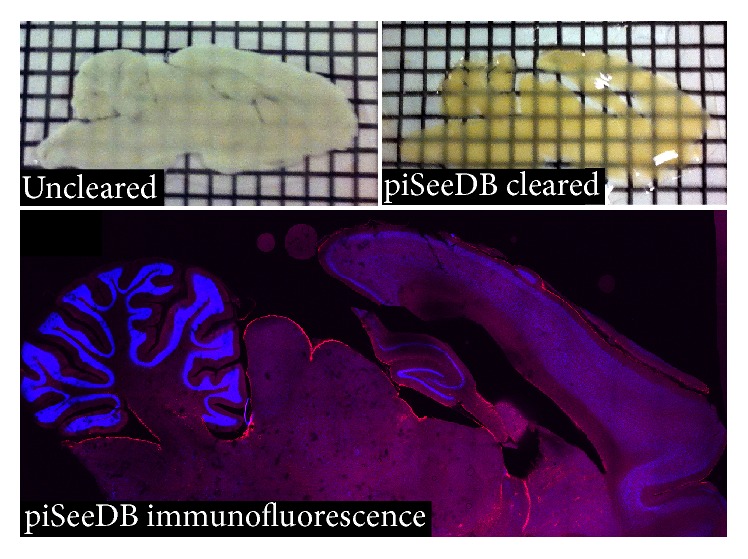
A comparison of the transparency of uncleared and piSeeDB cleared samples.

SeeDB was prepared utilizing procedures from Ke et al. [62]. Ascending concentrations of D-Fructose in PBS were prepared in the following w/v concentrations of D-Fructose in PBS: 20%, 40%, 60%, 80%, and 100%. The final solution of SeeDB was produced using 60.75 g of D-Fructose and 15 mL of distilled water. Solutions were heated to 60°C and stirred periodically until fully dissolved. After the solution reached RT, 300 *μ*L of *α*-thioglycerol was added in order to inhibit tissue browning from the Maillard reaction [62]. Tissue was then rotated in 20%, 40%, and 60% solutions for 8 hours each; 80% and 100% solutions for 12 hours each; and then SeeDB solution for 24 hours (Figure 1).

### 2.3. Imaging and Analysis

#### 2.3.1. Confocal Imaging

Tissue sections were placed in a SeeDB-filled imaging chamber composed of a pair of square cover slips with a 250 *μ*m silicon spacer placed between them. The sample was placed in its SeeDB solution in the interior of the imaging chamber with the most medial surface facing up. Confocal imaging was conducted with an Olympus Fluoview FV10i microscope in *z*-stack time-lapse mode. While piSeeDB was effective in clearing samples up to 2 mm in unparaffinized samples, and up to 250 *μ*m in deparaffinized samples, the working distance of the FV10i 60x oil-objective limited imaging to depths of roughly 100 *μ*m. This objective was used despite these limitations because high magnification is necessary to resolve the fine detail of astrocyte filaments. Images were captured with a region of interest centered against the anteriormost point of the granule cell layer of the dentate gyrus and measuring approximately 600 *μ*m (*x*), 400 *μ*m (*y*), and 100 *μ*m (*z*). Imaging duration was four days per sample. The sample was kept at 36°C during imaging.

#### 2.3.2. Image Processing and Analysis

Microscope images were produced as a *z*-series which was then stitched into *z*-stacks using XuvStitch 1.8.099. No compression was used. *z*-stacks were then visualized with Bitplane Imaris 7 (Bitplane, Concord, MA). *z*-stacks were normalized in order to maintain a consistent signal intensity through the depth of the sample.

**Figure 2:**
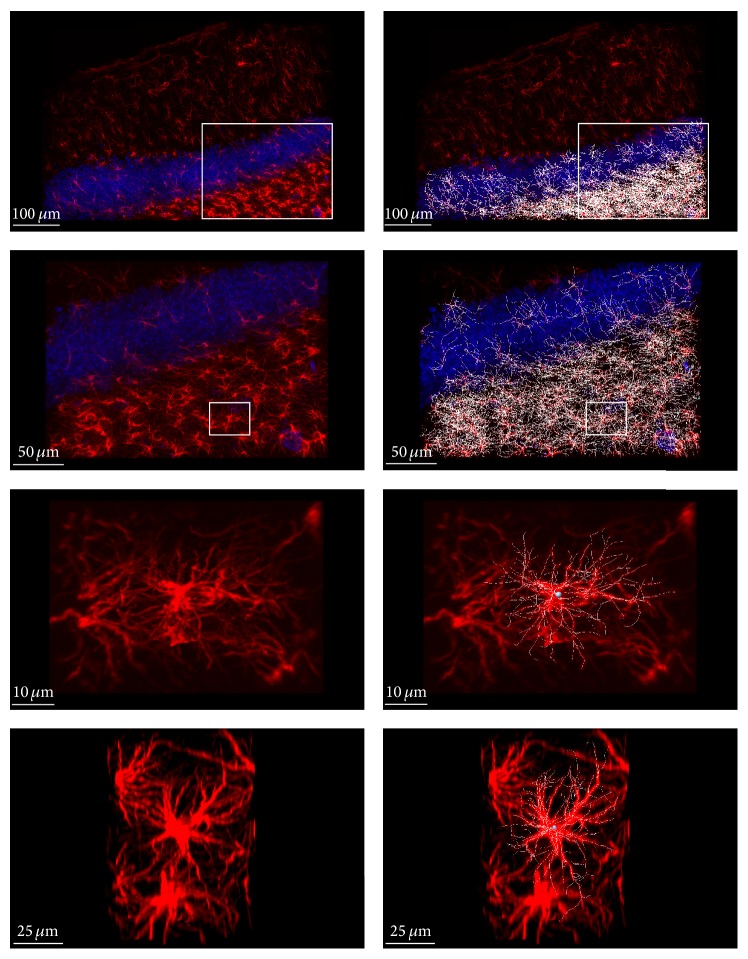
Astrocytes within the granule cell layer and hilus of the dentate gyrus were semiautomatically traced, segmented, and analyzed using Imaris FilamentTracer.

Astrocytes within the granule layer and the hilus of the dentate gyrus were analyzed. Imaris FilamentTracer was used with the AutoPath (no loops) algorithm to trace, segment, and statistically analyze astrocyte morphology (Figure 2). GFAP^+^ cells were counted automatically using a detection threshold value that was held constant between samples. An average of 171.88 GFAP^+^ cells was analyzed per sample. GFAP^+^ filaments were segmented and the length between branches, as well as the total length, was measured. Filament terminal points were then detected based on intensity thresholding. The number of branch points was also counted per cell. In addition, full branch depth, which measures the maximum number of branches between the starting point (nucleus) of the cell and a terminal point, was recorded. Finally, 3-dimensional Sholl analysis was used to measure filament complexity. The minimum, maximum, mean, and standard deviation per sample were analyzed for each measure of astrocyte morphology.

#### 2.3.3. Data Analysis

All data were analyzed with *t*-tests using SPSS 21.0 for Windows (IBM, Armonk, NY). Homogeneity of variance was verified using Levine's test. A *p* value of <.05 was considered significant for all statistical tests.

## 3. Results

The total number of GFAP^+^ cells within the granule cell layer and the hilus of the dentate gyrus were counted and analyzed. No significant differences were found in the number of GFAP^+^ cells between animals housed in EE (M = 200.25, SEM = ±14.56) and those housed in SE (M = 143.50, SEM = ±45.49) (*p* = .280). GFAP^+^ filament morphology was then analyzed to assess potential cellular differences between groups.

The total filament length was determined per cell and then averaged across samples. The mean total filament length was not significantly different between EE (M = 502.64, SEM = ±73.04) and SE (M = 361.00, SEM = ±23.10) (*p* = .146). The cell with the minimum total filament length was measured per sample and then averaged between groups. The mean minimum total filament length was not significantly different between EE (M = 38.2, SEM = ±19.10) and SE (M = 7.86, SEM = ±6.04) (*p* = .181). This analysis was repeated for cells with the maximum total filament length per sample and was also found to not be significant between EE (M = 1839.79, SEM = ±223.36) and SE (M = 1772.95, SEM = ±186.69) (*p* = .826). The standard deviation in total filament length was not significantly different between EE (M = 315.11, SEM = ±34.66) and SE (M = 297.34, SEM = ±15.86) (*p* = .658).

**Figure 3:**
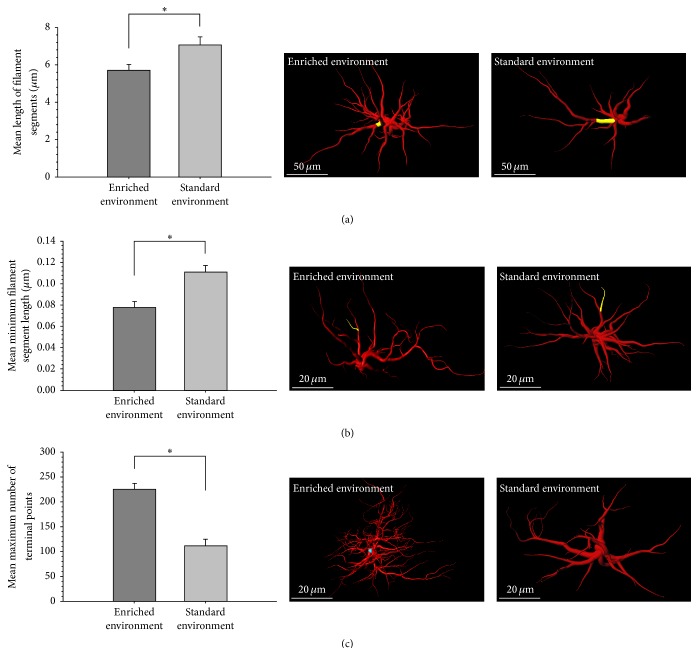
(a) Mean length of filament segments (regions between branch points), (b) mean minimum filament length, and (c) mean maximum number of terminal points. *∗* denotes a *p* value of <.05.

Within each cell, the mean length of GFAP^+^ individual filament segments was significantly shorter among EE (M = 5.71, SEM = ±0.31) than SE (M = 7.08, SEM = ±0.43) (*t*(6) = −2.576, *p* = .042, Figure 3(a)). In addition, the mean minimum length of individual GFAP^+^ filaments was significantly shorter among EE (M = 0.08, SEM = ±0.01) than SE (M = 0.11, SEM = ±0.01) (*t*(6) = −4.033, *p* = .007, Figure 3(b)). The mean maximum filament segment length was not significantly different between EE (M = 60.64, SEM = ±6.57) and SE (M = 82.19, SEM = ±10.42) (*p* = .131). The standard deviation in filament segment length was also not significant between EE (M = 10.68, SEM = ±5.44) and SE (M = 7.37, SEM = ±0.57) (*p* = .587).

The number of filament terminal points was also analyzed. The mean number of terminal points was not significantly different between EE (M = 48.08, SEM = ±8.63) and SE (M = 27.97, SEM = ±2.34) (*p* = .099). The mean maximum number of terminal points was significantly greater among EE (M = 225.75, SEM = ±26.02) than SE (M = 111.75, SEM = ±13.44) (*t*(6) = 3.893, *p* = .008, Figure 3(c)). The mean minimum number of terminal points was not significantly different between EE (M = 3.50, SEM = ±1.19) and SE (M = 0.75, SEM = ±0.48) (*p* = .100). The standard deviation in the number of terminal points was also not significantly different between EE (M = 26.95, SEM = ±9.18) and SE (M = 19.82, SEM = ±2.50) (*p* = .501).

**Figure 4:**
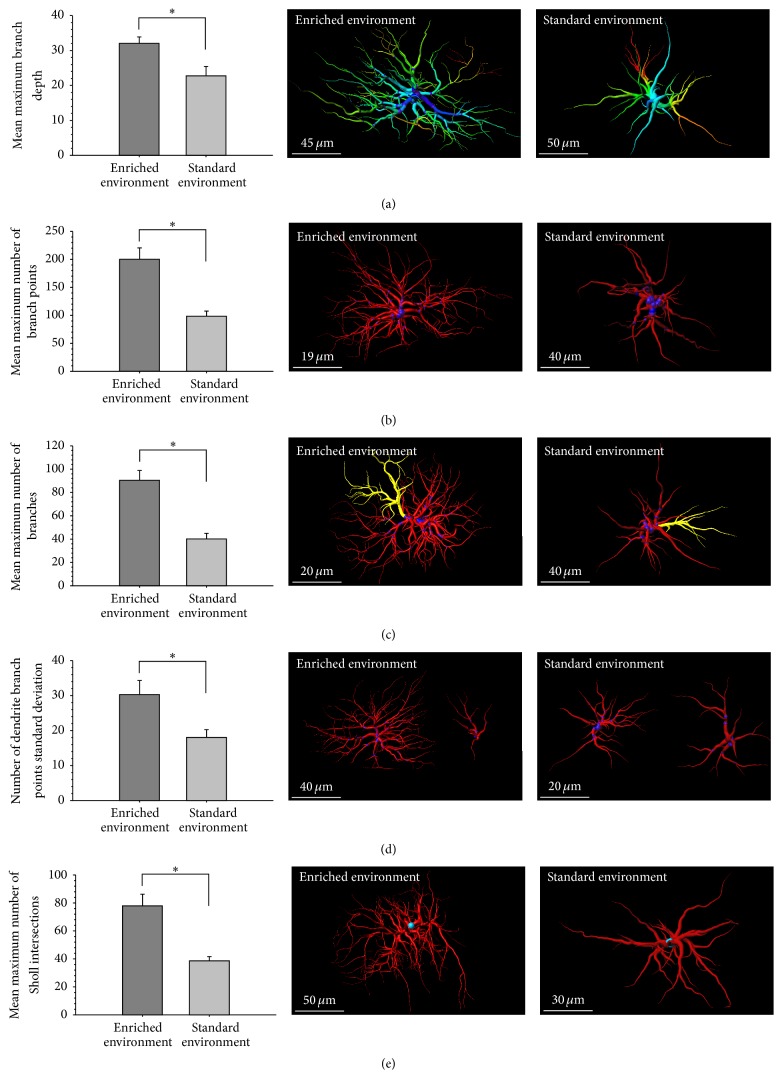
(a) Mean maximum branch depth. (b) Mean maximum number of branch points. (c) Mean maximum number of branches. (d) Standard deviation in the number of branch points. (e) Mean maximum number of Sholl intersections. *∗* denotes a *p* value of <.05.

The total number of branch points was calculated per cell. The mean total number of branch points per cell was not significantly different between EE (M = 42.00, SEM = ±8.26) and SE (M = 23.60, SEM = ±2.17) (*p* = .109). The minimum and maximum number of branch points per cell were averaged across samples and compared across groups. The maximum number of branch points was significantly greater among animals housed in EE (M = 200.50, SEM = ±20.52) than SE (M = 99.25, SEM = ±9.14) (*t*(6) = 4.508, *p* = .004, Figure 4(b)). The standard deviation in the number of branch points per cell was also calculated between groups and was significantly greater among EE animals (M = 30.38, SEM = ±3.92) compared to SE animals (M = 18.19, SEM = ±2.09) (*t*(6) = 2.743, *p* = .045, Figure 4(d)). The mean number of branches was not significantly different between EE (M = 11.41, SEM = ±1.88) and SE (M = 7.72, SEM = ±0.82) (*p* = .109). The maximum number of branches was significantly greater among samples from the EE group (M = 90.75, SEM = ±8.29) compared to the SE group (M = 40.50, SEM = ±4.79) (*t*(6) = 5.249, *p* = .002, Figure 4(c)).

The full branch depth (the greatest number of branches taken to reach a terminal point) was measured per cell and then averaged per sample and compared across groups. The value for the cell with the greatest full branch depth per sample was averaged across samples. Among the EE group, cells with the maximum branch depth had significantly more branches (M = 32.00, SEM = ±1.87) than those in the SE group (M = 22.75, SEM = ±2.72) (*t*(6) = 2.802, *p* = .031, Figure 4(a)). The cell with the lowest full branch depth per sample was also averaged across samples; however there were no significant differences when compared between EE (M = 1.50, SEM = ±0.87) and SE (M = 0.50, SEM = ±0.29) (*p* = .315). There were no significant differences in the mean full branch depth between EE (M = 9.47, SEM = ±1.55) and SE (M = 8.16, SEM = ±0.50) (*p* = .453). There were also no significant differences in standard deviation of full branch depth between EE (M = 4.19, SEM = ±0.23) and SE (M = 3.91, SEM = ±0.41) (*p* = .573).

Astrocyte filament complexity was further analyzed using three-dimensional Sholl analysis. The maximum number of Sholl intersections per sample was averaged across groups and found to be significantly greater among EE animals (M = 78.00, SEM = ±8.33) compared to SE animals (M = 38.75, SEM = ±2.95) (*t*(6) = 4.438, *p* = .004, Figure 4(e)). There were no significant differences in the overall mean number of Sholl intersections between EE (M = 10.16, SEM = ±1.49) and SE (M = 7.11, SEM = ±0.67) (*p* = .111). In addition, the standard deviation of Sholl intersections was not significantly different between EE (M = 14.50, SEM = ±4.55) and SE (M = 6.41, SEM = ±0.58) (*p* = .128).

## 4. Discussion

It was hypothesized that enriched environments would induce an increase in the number and complexity of astrocytes in the rat dentate gyrus. However, evidence was not found for an increase in the number astrocytes, in contrast to results from other studies in different brain regions such as the visual cortex [50]. This may be due to a difference in the mechanisms mediating plasticity in different brain regions. The possibility that astrocytes respond to EE through changes in morphology rather than number of cells was also investigated. The results indicated a significantly shorter distance between branching points but no differences in the overall number of Sholl intersections, terminal points, branch number, or total filament length. However, a more detailed analysis demonstrated significant differences between cells with the most complex filaments when compared between EE and SE. The cells with the maximum value per sample for the number of Sholl intersections, number of terminal points, and number of branches per cell were significantly larger among astrocytes from EE animals. These data suggest that astrocytes of the dentate gyrus may not be uniformly affected by EE. Recent work has revealed that astrocyte populations are genetically and morphologically heterogeneous, even within individual brain regions [64, 65], a finding that may explain the differential effects of EE on astrocyte morphology. Furthermore, it is possible that the effect of EE on astrocytes is limited primarily to fully mature astrocytes, which are likely to exhibit a greater degree of filament complexity relative to more immature astrocytes.

An increase in astrocyte process complexity due to enriched housing environments may substantially alter the ability of affected astrocytes to influence neuronal properties within their domains. In response to EE, neurons form new dendritic spines as well as modulating the structure and function of existing spines [20–22]. As a critical regulator of spine dynamics, astrocytes are likely to be playing a key role in this process [36–41]. Increased astrocyte filament complexity in response to EE lends further support to this hypothesis. A more detailed understanding of how astrocytes affect plasticity will require further research of the interactions between newly formed astrocyte processes and the properties of the synapse in the context of environments of varying complexity.

It is possible that morphological changes in astrocytes in response to EE may not be fully evident by merely measuring changes in GFAP expression. Recent studies have demonstrated that as much as 85% of astrocyte volume is not shown by GFAP labeling [27]. In addition, not all astrocytes express GFAP at significant levels, despite meeting the other qualifications for astrocyte identity [66]. Thus, GFAP labeling may fail to capture smaller scale changes in astrocyte complexity. Nevertheless, these results suggest the possibility that EE invokes an increase in GFAP filament complexity among a subset of dentate gyrus astrocytes. New advances in molecular identification of astrocytes will be an important step in understanding the heterogeneous plasticity of astrocytes observed in this study.

Alteration of astrocyte structure and function is a hallmark feature of numerous neurological diseases including neurodegenerative diseases such as Alzheimer's Disease [56–59] and Parkinson's Disease [60, 61], as well as traumatic brain injury [67–69], stroke [70–73], and epilepsy [52–55, 74–76]. Activated astrocytes in these diseases undergo reactive gliosis, which involves the upregulation of astrocyte GFAP, as well as breakdown of domain specificity [64]. In TBI, serum levels of GFAP have been proposed as a potential biomarker of disease severity [68, 69]. As the results from this study indicate that astrocytes from animal models housed in SE may exhibit pathologic GFAP filament morphology, it is crucial to understand what effect housing environment may have on disease recovery processes and on the results of studies using animal models in general.

Despite these results, the potential role of astrocytes in mediating the behavioral effects of EE remains unclear. Detailed analysis of astrocyte morphological, electrophysiological, and genetic dynamics in the context of EE is crucial for understanding how plasticity in the dentate gyrus and other brain regions is controlled. New optical clearing techniques such as piSeeDB will be critical in understanding these processes. Despite recent advances in histological methods which allow imaging of large three-dimensional sections of tissue, specialized software for the automated analysis of* in situ* morphology is limited, and analysis by hand is extremely time-consuming, especially when dealing with large samples. New advances in automatic three-dimensional morphology analysis as well as in processing and storage of large image data sets are thus critically needed for the study of complex brain anatomy and physiology.
